# Decreasing Poly(ADP-Ribose) Polymerase Activity Restores ΔF508 CFTR Trafficking

**DOI:** 10.3389/fphar.2012.00165

**Published:** 2012-09-12

**Authors:** Suzana M. Anjos, Renaud Robert, Daniel Waller, Dong Lei Zhang, Haouaria Balghi, Heidi M. Sampson, Fabiana Ciciriello, Pierre Lesimple, Graeme W. Carlile, Julie Goepp, Jie Liao, Pasquale Ferraro, Romeo Phillipe, Françoise Dantzer, John W. Hanrahan, David Y. Thomas

**Affiliations:** ^1^Cystic Fibrosis Translational Research Center, Department of Biochemistry, McGill UniversityMontreal, QC, Canada; ^2^Cystic Fibrosis Translational Research Center, Department of PhysiologyMontreal QC, Canada; ^3^Division of Thoracic Surgery, Centre Hospitalier de l’Université de MontréalMontréal, QC, Canada; ^4^Department of Pathology, Montreal Heart Institute, Université de MontréalMontréal, QC, Canada; ^5^University of StrasbourgIllkirch, France

**Keywords:** CF, Cystic fibrosis, ABT-888, PARP-1, oxidative stress, DNA damage PARP-1^−/−^

## Abstract

Most cystic fibrosis is caused by mutations in CFTR that prevent its trafficking from the ER to the plasma membrane and is associated with exaggerated inflammation, altered metabolism, and diminished responses to oxidative stress. PARP-1 is activated by oxidative stress and causes energy depletion and cell dysfunction. Inhibition of this enzyme protects against excessive inflammation and recent studies have also implicated it in intracellular protein trafficking. We hypothesized that PARP-1 activity is altered in CF and affects trafficking and function of the most common CF mutant ΔF508 CFTR. Indeed, PARP-1 activity was 2.9-fold higher in CF (ΔF508/ΔF508) human bronchial epithelial primary cells than in non-CF cells, and similar results were obtained by comparing CF vs. non-CF bronchial epithelial cell lines (2.5-fold higher in CFBE41o^−^ vs. 16HBE14o^−^, *P* < 0.002). A PARP-1 inhibitor (ABT-888, Veliparib) partially restored CFTR channel activity in CFBE41o^−^ cells overexpressing ΔF508 CFTR. Similarly, reducing PARP-1 activity by 85% in ileum from transgenic CF mice (*Cftr^tm1^*Eur**) partially rescued ΔF508 CFTR activity to 7% of wild type mouse levels, and similar correction (7.8%) was observed *in vivo* by measuring salivary secretion. Inhibiting PARP-1 with ABT-888 or siRNA partially restored ΔF508 CFTR trafficking in cell lines, and most ΔF508 CFTR was complex glycosylated when heterologously expressed in PARP-1^−/−^ mouse embryonic fibroblasts. Finally, levels of the mature glycoform of CFTR were reduced by peroxynitrite, a strong activator of PARP-1. These results demonstrate that PARP-1 activity is increased in CF, and identify a novel pathway that could be targeted by proteostatic correctors of CFTR trafficking.

## Introduction

Cystic fibrosis is the most prevalent inherited disease amongst Caucasians, afflicting ∼70,000 people worldwide (Riordan et al., 1989). The symptoms of cystic fibrosis include progressive respiratory dysfunction due to persistent and repeated cycles of infection and inflammation, and are caused by mutations in the cystic fibrosis transmembrane conductance regulator (CFTR) gene. CFTR encodes an ATP-binding cassette (ABC) transporter that functions as a tightly regulated anion channel. Over 1,900 mutations in CFTR have been identified, the most prevalent being an in-frame deletion of Phe at the 508 position (ΔF508; Bobadilla et al., 2002). This mutation, which is present on at least one chromosome in 90% of people with CF, causes the mutant protein to be recognized by the cellular quality control machinery and retained at the ER where it is then degraded (Cheng et al., 1990). ΔF508 also reduces the open probability of mutant channels that reach the plasma membrane shortening their half-life at the cell surface (Lukacs et al., 1994). Although the life expectancy for CF patients has improved in recent years due to improved antibiotics, pancreatic enzyme supplements, and therapeutic regimens, there remains no cure for most people with CF who carry CFTR mutations that cause defective trafficking.

Cystic fibrosis transmembrane conductance regulator mutations cause a myriad of downstream biological changes, and the relationship between these changes and the disease phenotype remains poorly understood. Markers of oxidative stress are elevated in the plasma, presumably due to pulmonary infection (Brown et al., 1996; Collins et al., 1999). CF patients also display increased susceptibility to oxidative-induced DNA damage as measured by urinary excretion of 8-hydroxydeoxyguanosine, and this sensitivity to oxidants may be an inherent property of the disease since it appears to be independent of clinical status (Brown et al., 1995). High intracellular levels of hydrogen peroxide and mitochondrial reactive oxygen species (ROS) have been reported in CFTR-deficient cells (Rottner et al., 2009), and a deficiency in reduced glutathione (GSH) in the respiratory epithelial lining fluid and plasma has been known for some time (Roum et al., 1993). Thus several lines of evidence suggest that CF leads to redox disturbances, as recently reviewed (Galli et al., 2012). One mechanism used by cells to protect against oxidative DNA damage is PolyADP (Ribose) Polymerase-1 (PARP-1), the most abundant isoform of a family of nuclear enzymes that sense DNA damage and initiate DNA repair. PARP-1 is activated by cell stress and plays an important role during tissue injury (Luo and Kraus, 2005; Pacher and Szabo, 2008). It uses NAD^+^ to transfer polymers of ADP-ribose to target proteins at the expense of ATP, a post-translational modification known as poly ADP-(ribosyl)ation (PARylation). PARP-1 function depends on the type, duration and strength of the stress stimuli, and on the proliferative and metabolic state of the cell (Luo and Kraus, 2005), and is intimately tied to nuclear NAD+ metabolism and the broader cellular metabolic profile (Luo and Kraus, 2005).

Under conditions of cell stress the nuclear enzyme poly(ADP-ribose) polymerase-1 (PARP-1) becomes hyperactive and depletes cells of NAD^+^. This slows glycolysis, reduces electron transport and ATP formation, and may lead to the upregulation of proinflammatory pathways or cell death (Cuzzocrea, 2005; Pacher and Szabo, 2008). Thus, from a pathophysiological standpoint, PARP activation could contribute to disease by driving the cell into an energetic deficit, and also by inducing a state of dysfunction through activation of proinflammatory pathways (Cuzzocrea, 2005). Both these mechanisms have been implicated in CF. We hypothesize that the misfolded mutant CFTR is associated with an increase in PARP activity. Decreasing this activity may restore some functional correction.

In this study we have investigated a possible role of PARP-1 in the regulation of ΔF508 CFTR trafficking and function in CF bronchial epithelial cells. The involvement of PARP-1 in oxidative stress, inflammatory responses, and energy maintenance, and its emerging role as a regulator of intracellular protein trafficking (Abd Elmageed et al., 2011) suggested that it may be an interesting potential target for small molecule correctors in Cystic fibrosis.

## Materials and Methods

### Reagents

4-Amino-1,8-naphthalimide (4-AN) and N-(6-Oxo-5,6-dihydrophenanthridin-2-yl)-(N,N-dimethylamino)acetamide hydrochloride (PJ34) were obtained from Sigma-Aldrich (Oakville Ontario, Canada). (2-((2R)-2-Methylpyrrolidin-2-yl)-1H-benzimidazole-4-carboxamide dihydrochloride; ABT-888) was purchased from Alexis Biochemicals (Famingdale, NY, USA). VRT-325 was a generous gift from Dr. Robert Bridges (Rosalind Franklin University of Medicine and Science) and the Cystic Fibrosis Foundation Therapeutics Inc. (CFFT). The monoclonal antibody against the R domain of CFTR (clone 23C5) was generated by our lab (Myriam Mirza, Veli-Pekka Määttänen and D. Y. T., unpublished data). M3A7 monoclonal antibody against CFTR was purchased from Chemicon (Billerica, MA, USA). α-tubulin was obtained from Sigma-Aldrich and monoclonal antibody (IgG2_a_) against PARP-1 was from Santa Cruz Biotechnology (Santa Cruz, CA, USA). Anti-PAR was obtained from Trevigen (Gaithersburg, MD, USA). Rabbit anti-hERG antibody was obtained from Calbiochem (Burlington, Ontario, Canada). Peroxynitrite (tetramethylammonium) was obtained from Alexis Biochemicals and prepared in ice cold 0.01 M KOH as per the manufacturer’s instructions.

Homozygous Δ508 CFTR mice (*Cftr^tm1^*Eur**; van Doorninck et al., 1995) and wild type littermates controls were used in the *ex vivo* experiments. Breeders and protocols for mouse intestinal assays were kindly provided by B. J. Scholte, M. Wilke, and H. R. de Jonge, Erasmus University Medical Center, Rotterdam, NL. The mice were kept in the animal facility at McGill University and fed a high protein diet (SRM-A, Hope Farms, Woerden, Netherlands) modified to contain pork instead of beef. All procedures followed Canadian Institutes of Health Research (CIHR) regulations and were approved by the faculty Animal Care Committee of McGill University.

### Cell culture and treatments

HEK293 cells were stably transfected with HA tagged hERG G601S (generous gift of E. Ficker; Wible et al., 2005) and maintained in standard culture conditions.

Stably transfected BHK cells expressing histidine-tagged (His) wt-CFTR or ΔF508 CFTR were maintained in DMEM-F12 media supplemented with 5% FBS and 450 μM methotrexate. 1% l-Glutamax. CFBE41o^−^ cell lines transduced with TranzVector lentivectors containing ΔF508 CFTR (CFBE41o-ΔF508) and wild type CFTR (CFBE41o^−^CFTR) cells were kindly provided by J. P. Clancy (Bebok et al., 2005) and were maintained in EMEM (Wisent, St-Bruno, QC, USA) supplemented with 10% FBS and 1% l-Glutamax. For polarized CFBE41o^−^ monolayers (ΔF508 and wt-CFTR), cells were cultured under liquid/liquid conditions and polarized at the air/liquid interface. Cells were seeded at a density of 2.5 × 10^5^ cells/cm^2^ onto 12 mm fibronectin-coated Snapwell inserts (Corning Incorporated). The apical medium was removed after 24 h to establish an air-liquid interface (ALI), and then the cells were cultured for another 6–7 days (Bebok et al., 2005). CFBE41o^−^ cells were treated with 4-AN (Sigma-Aldrich), PJ34 (Sigma-Aldrich), or ABT-888 (Alexis Biochemicals) for 24 h or as shown and at the indicated concentrations. Low temperature rescue was carried out at 29°C for 24 h or as indicated. DMSO was used as a vehicle at a 1:1000 dilution. Primary Human Bronchial Epithelial cells (HBEs) were isolated from human bronchial tissue by enzyme digestion and cultured in bronchial epithelial growth medium (BEGM; Fulcher et al., 2005) on vitrogen-coated plastic flasks (Vitrogen 100, PureCol, Advanced BioMatrix #5005-B). They were then trypsinized, counted, and cryopreserved or transferred onto collagen VI-coated snapwell culture inserts (Corning, catalog no. 3801) in ALI medium (Fulcher et al., 2005) at a density of 2.5 × 10^5^ cells/insert. During the first 4 days, the ALI medium was changed daily, then apical media was removed and the cells were grown in an ALI for 22 days before use. For the CF HBE cells, the isolation and growth media were complemented with specific antibiotics based on the patient’s microbiology report.

### Immunoblotting and densitometry

BHK cells overexpressing (His) ΔF508 CFTR and wt-CFTR, and CFBE41o^−^ cells (overexpressing ΔF508 and wt-CFTR) were lysed in RIPA buffer containing protease inhibitors (Roche, Inc.) and 0.8% deoxycholic acid prepared as described (Robert et al., 2008). Briefly, 10 μg (BHK) and 20 μg (CFBE41o^−^) total protein were separated using 6% (v/v) SDS-PAGE and transferred onto nitrocellulose membranes. Membranes were probed with monoclonal anti-CFTR antibody (clone 23C5) at a 1:10 dilution overnight at 4°C for CFBE41o^−^ lysates or 1:1000 dilution for BHK lysates (clone M3A7). Membranes were reprobed for PARP1 with monoclonal antibody against PARP1 at a 1:500 dilution. For immunoblotting against hERG in the HEK293 cells, polyclonal antibody against hERG was used overnight at 4°C at 1:1000 dilution. Membranes were probed with monoclonal anti-tubulin (Sigma-Aldrich) to normalize for protein loading. The relative levels of each CFTR glycoform were estimated by densitometry using the Image J program (http://rsb.info.nih.gov/ij/). The relative amount of band B or band C is calculated as a fraction of tubulin for the respective lane and reported as a fraction of the total (band C/tub + band B/tub). The values reported are expressed as means ± SD (*n* = 3). Data sets were compared by a Student’s *t*-test using SigmaPlot (Systat Software, Inc.).

### Halide flux assay and voltage clamp studies of CFBE41o^−^ cell monolayers

Iodide efflux from BHK cells expressing (His) ΔF508 CFTR was assayed using a robotic liquid handling system (BioRobot 800 Qiagen, USA) and Qiagen 4.1 software. Iodide concentration at the end of each sample period was measured using an iodide-sensitive electrode (Orion Research, Inc., Boston, MA, USA) and converted to iodide content released per 1 min interval. Relative iodide efflux rates were calculated by subtracting the baseline from the peak iodide flux (in μmol/min). Data were calculated from at least three independent experiments each with four replicates, and are reported as ±SEM. Short-circuit current (*I*_sc_) was measured across monolayers mounted in modified Ussing chambers. CFBE41o^−^ cells over expressing ΔF508 CFTR and wt-CFTR (250 000) were seeded onto 12-mm fibronectin-coated Snapwell inserts (Corning Incorporated) and the apical medium was removed after 24 h to establish an ALI. CFBE41o^−^ ΔF508 monolayers were treated on both sides with Opti-MEM medium (no FBS) and one of the following compounds: 0.1% DMSO (negative control), 1 nM ABT-888, 10 μM VRT325. Alternatively, CFBE41o^−^ΔF508 cells were incubated at 29°C (positive control) for 24 h before being mounted in chambers and voltage clamped using a VCCMC6 multichannel current-voltage clamp (Physiologic Instruments, San Diego, CA, USA). The assay was performed as described previously (Robert et al., 2008).

### PARP1^*−/−*^ and PARP1^+*/*+^ mouse embryonic fibroblasts

PARP1*^−/−^* and PARP1^+/+^ MEFs were obtained from Françoise Dantzer (CR1, CNRS, University of Strasbourg, France) and maintained in DMEM supplemented with FBS (10%) and gentamicin (1%) at 37°C, 5% CO_2_. The cells were transfected with wild type or ΔF508 triple-HA tagged CFTR in pcDNA3.1 plasmid using Fugene according to the manufacturer’s guidelines. Briefly, 2 × 10^5^ cells were seeded in a 6-well plate and transfected when at least 50% confluent using 7 μl Fugene: 2 μg DNA. For immunoblotting, 40–50 μg of total protein was loaded into each lane and membranes were probed with monoclonal antibodies against CFTR and tubulin (see above).

### PARP activity assays

Approximately 5 × 10^4^/200 μl CFBE41o^−^ cells were cultured in 96 well plates and treated with ABT-888 at the indicated concentrations for 24 h. Cells were then lysed buffer (supplied in kit) to which 4 mM NaCl, 1% Triton X-100, and 200 μM PMSF are added. Total protein is quantified by the Bradford assay and 25 μg total protein per well was assayed in triplicate. PARP activity was measured in histone-coated strip wells using the High Throughput (HT) Chemiluminescent PARP/Apoptosis Assay (Trevigen) following the manufacturer’s procedures. This ELISA measures the incorporation of biotinylated poly(ADP-ribose; PAR) into histone proteins by chemiluminescence after samples are incubated with anti-PAR antibody and then HRP-conjugated secondary antibody (anti-mouse IgG-HRP). Readings were taken using an HT Analyst Criterion Host, and the light output was proportional to PARP-1 activity. PARP activity for each sample is calculated from a standard curve ran in triplicates within each experiment. Results are expressed in mUnits PARP activity/μg of total protein.

*In vitro* PARP-1 activity was measured HT Universal Chemiluminescent PARP Assay (Trevigen) following the manufacturer’s instructions.

Inhibitors are identified when a PARP mediated increase in fluorescence signal indicating the accumulation of NAD^+^ (or the decrease in the PARP mediated depletion of NAD^+^). In the absence of PARP maximal signal is observed, whereas in its presence minimal signal is observed and% inhibition is calculated: % inhibition = 100 x [NAD remaining] for inhibitor at specific concentration/[NAD remaining] no inhibitor.

Since DMSO inhibits inhibitors were tested by serial dilution in water. Readings were taken automatically by the HT Analyst Criterion Host.

### siRNA silencing of *PARP1*

siRNAs (smart pool) were obtained from Thermo Scientific Dharmacon (Lafayette, CO, USA). siRNA was transfected using the NHBE Nucleofector kit (Lonza, Walkersville, USA), program W-001. *siPARP1/2* (200 nM) or scrambled siRNA (200 nM) was used per 10 cm plate of CFBE41o^−^ cells overexpressing ΔF508 CFTR. Following nucleofection, cells plated in fibronectin-coated 6-well plates in Opti-MEM overnight. The following day the medium was replaced with EMEM supplemented with 1% penicillin/streptomycin, 1% glutamate, and 10% FBS. Cells were lysed and total protein quantified by the Bradford assay. Immunoblots were probed for CFTR and PARP1 to assess trafficking and protein knockdown, respectively, as described above.

### *Ex vivo* assays of ABT-888 on CFTR-dependent current

Mice were genotyped by RT-PCR using tail DNA and used for experiments between age 14–17 weeks (24–30 g). Homozygous Δ508 CFTR mice (*Cftr^tm1^*Eur**; van Doorninck et al., 1995) and wild type littermates controls were used in the *ex vivo* experiments. For *ex vivo* experiments, the mucosa from the distal third of the ileum was stripped of muscle and mounted in Ussing chambers as described previously (Robert et al., 2008). Indomethacin (10 μM) was added to both sides to block prostaglandin synthesis and 10 μM amiloride was added apically to inhibit electrogenic Na^+^ absorption. CFTR-dependent *I*_sc_ was measured after 10–15 min equilibration, then 10 μM forskolin and 50 μM genistein were added (0 h). Both sides were then exposed to 1 nM ABT-888 or vehicle (distilled water) for 4 h, and the *I*_sc_ response to forskolin + genistein was measured again. Results are expressed as the mean ± SEM of *n* pieces of ileum from 5 wt-CFTR and 5 ΔF508 mice.

### Salivary secretion

The procedure followed those described by Best and Quinton (Best and Quinton, 2005). Homozygous ΔF508 CFTR mice (*Cftr^tm1^*Eur**; French et al., 1997) and wild type littermates were used at 10–12 weeks, 20–25 g. They were injected intraperitoneally with saline containing ABT-888 (5 mg/kg/day) or vehicle alone (saline) for 2 days. Details of the procedure have been described (Robert et al., 2008). Results are expressed as the mean ± SEM of *N* mice.

## Results

### Elevated PARP-1 activity in human CF bronchial epithelial cells

**Figure 1:**
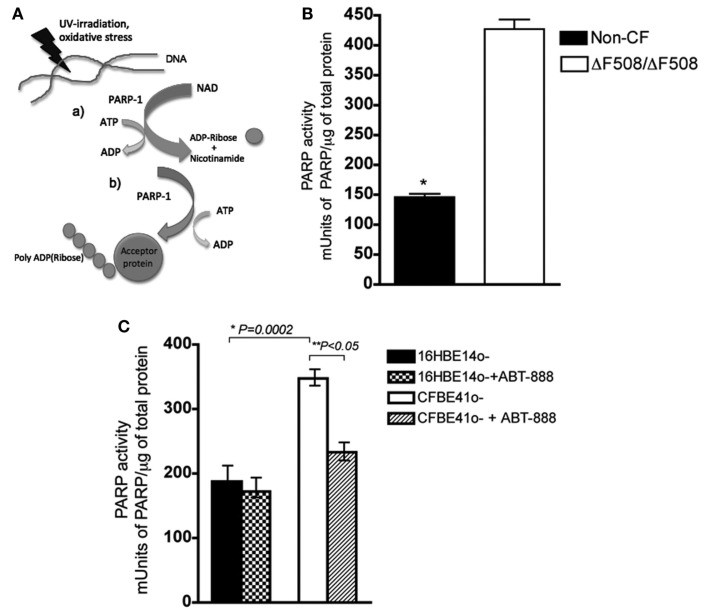
**PARP activity is elevated in CF primary and transformed epithelial cells and ABT-888 decreases PARP and activity in CF cells**. **(A)** (a) PARP-1 cleaves NAD^+^, releasing ADP-Ribose at the expense of ATP (b) PARP-1 synthesizes polymers of ADP-Ribose onto acceptor proteins **(B)** PARP-1 activity was measured in primary lung epithelial cells from two CF patients and non-CF donors. Activity is reported as mUnits of PARP-1/total protein (μg), where 1 unit is defined as the amount of PARP-1 that incorporates 100 pmol poly (ADP-ribose) from NAD^+^ into an acid-insoluble form. Bar graph shows the mean ± SD from *n* = 3 measurements per patient, and *denotes a significant difference at a *P* < 0.05 (two-tailed Student’s *t*-test). **(C)** PARP-1 activity is higher in the CFBE41o^−^ (CF) cell line than in the 16HBE14o^−^ (non-CF) cell line. PARP-1 activity is reduced by ∼40% in CFBE cells upon treatment with the PARP-1 inhibitor ABT-888 at the 1 nM (24 h) but ABT-888 has no effect on PARP-1 activity in the non-CF 16HBE14o^−^. Bar graph represents the mean ± SEM from *n* = 5 with three replicates each. Results are considered significant at *P = 0.0002 and **P < 0.05, as assessed by the paired, two-tailed Student’s *t*-test.

We measured PARP-1 activity in CF and non-CF primary HBEs by using ELISA to determine the rate at which its substrate NAD^+^ is assembled into polymers of ADP-Ribose as illustrated in Figure 1A. PARP-1 activity was 2.9-fold higher in HBEs from two CF patients when compared with cells from two non-CF subjects (Figure 1B, Students *t*-test, *P* < 0.05). Since primary HBEs may vary due to differences in genetic background and perhaps previous infection and inflammation history, PARP-1 activity was also measured in the commonly used CF and non-CF cell lines CFBE41o^−^ and 16HBE14o^−^, respectively, which express only endogenous mutant or wild type CFTR at low levels. PARP-1 activity was also higher in the CF cell line compared with the non-CF line (Figure 1C, 2.5-fold, *n* = 6, Students’ *t*-test, *P* = 0.002), as observed in primary HBEs. PARP-1 activity in CFBE41o^−^ cells was sensitive to ABT-888 (Veliparib) at 1 nM, decreasing PARP-1 activity ∼40% (*P* < 0.05), but not reducing it to wild type levels. ABT-888 is a potent inhibitor of PARP-1 in other cells systems, where it has an IC_50_ = 5 nM (Donawho et al., 2007). We treated wild type cells with ABT-888 (1 nM) and found that we could not abolish PARP-1 activity (Figure 1C), in agreement with previous evidence that some baseline PARP-1 activity is required for the maintenance of genomic stability (Luo and Kraus, 2005).

### Modulating PARP-1 activity restores chloride function

**Figure 2:**
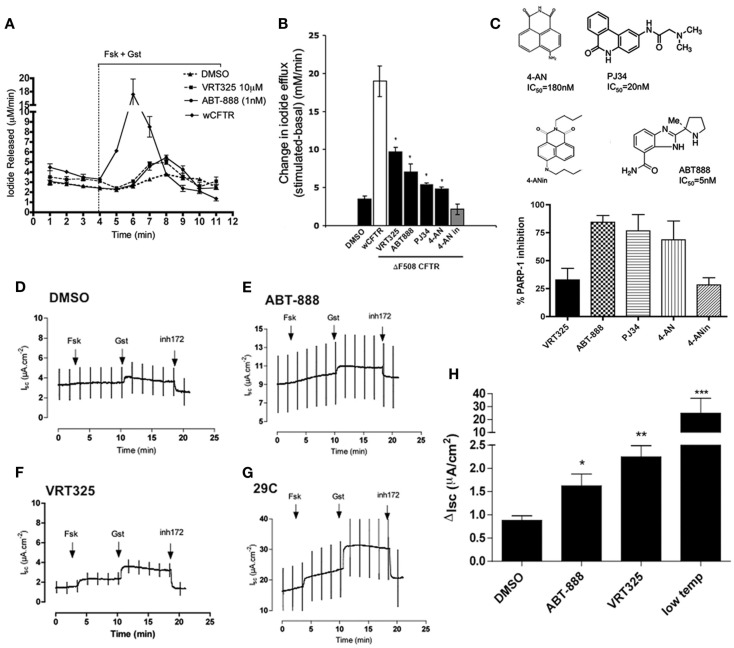
**PARP-1 inhibitors partially restore chloride activity in (BHK) CFTR ΔF508 and in polarized CFBE41o^−^ (ΔF508) cells. (A)** Iodide efflux trace measuring ΔF508 CFTR function at the plasma membrane in BHK cells treated with ABT-888 (1 nM), DMSO (0.1%), and VRT325 (10 μM) for 24 h. Iodide efflux trace of BHK cells expressing wild type (wt) CFTR is also shown. An arrowhead and bar graph indicate addition and maintenance, respectively, of 10 μM forskolin (Fsk) and 50 μM genistein (Gst). Error bars indicate ±SD (*n* = 3). Note break in axis **(B)** change in iodide efflux upon stimulation with Fsk and Gst defined as the peak iodide efflux after stimulation subtracted from the baseline response summarizing data from all compounds tested; 4-AN (0.1 μM), 4-ANin (0.1 μM), PJ34 (10 μM), ABT-888 (1 μM). VRT-325 (10 μM) and DMSO (0.1%). Error bars indicate ±SEM (*n* = 3). A difference in the means as compared with the DMSO control was tested for statistical significance using paired *t*-tests (*P < 0.05). **(C)** Chemical structures of the PARP-1 inhibitors and the inactive 4-AN their published IC_50_ (PARP-1 inhibition) are shown. The extent of PARP-1 inhibition (*in vitro*) was determined for each of the compounds by measuring the NAD^+^ remaining in the presence of inhibitor. Results are reported as %inhibition of PARP-1. VRT325, as expected is not a PARP-1 inhibitor and 4-ANin, an inactive analog of 4-AN, is also not an inhibitor. ABT-888 is the most potent PARP-1 inhibitor we tested. The graph summarizes the data from three independent experiments representing the mean ± SEM. **(D–G)** Four panels show polarized CFBE41o^−^ cells stably overexpressing ΔF508 CFTR treated with DMSO (0.1%), ABT-888 (1 nM), VRT325 (10 μM), and low temperature incubation (29°C) for 24 h and current was measured in Ussing chamber. CFTR-mediated currents cause upward deflections because an apical to basolateral current was imposed after permeabilization of the basolateral membrane. **(H)** Bar graph showing the change in *I*_sc_ (disk) after the addition of 10 μM Fsk and 50 μM Gst, defined as the difference between the sustained current after genistein and baseline arrest before stimulation. Error bars represent mean ± SEM (*n* = 4) at (*P < 0.05), (**P < 0.001), or (***P < 0.0001) relative to DMSO (paired *t*-tests). Note break in *y*-axis.

To examine the influence of PARP-1 on CFTR channel activity, iodide (I^−^) efflux was measured from BHK cells overexpressing ΔF508 CFTR after pretreatment with PARP-1 inhibitors for 24 h. Cells were pretreated with inhibitor, loaded by incubation in iodide solution for 1 h, then stimulated acutely with 10 μM forskolin (Fsk) in combination with the potentiator 50 μM genistein (Gst). Pretreating cells with a well characterized corrector VRT325 (10 μM, 24 h) increased the iodide efflux threefold compared to the DMSO vehicle control (Figures 2A,B, *P* < 0.05, Student’s *t*-test), consistent with a previous report (Loo et al., 2005). Pretreatment with ABT-888 increased iodide efflux (2.7-fold above DMSO; Figure 2B, *n* = 3, Students’ *t*-test, *P* < 0.05) to levels comparable to VRT325. We also tested other PARP-1 inhibitors with different structures (though all share the same carbonyl group), potencies and mechanisms of action (see Figure 2C for structures), and an inactive analog of 4-AN (4-ANin; Figures 2B,C). Both PJ34 and 4-AN increased forskolin + genistein stimulated iodide release from BHK cells stably expressing ΔF508 CFTR, suggesting partial rescue of functional channels to the cell surface representing approximately half of the response obtained with VRT325. The inactive analog (4-ANin) that does not inhibit PARP-1 had no effect (Figure 2B). We monitored the *in vitro* PARP-1 activities of the inhibitors tested and found that their relative ability to inhibit PARP-1 correlated with their ability to restore iodide release in (BHK) ΔF508 CFTR (graph, Figure 2C) suggesting that their effects are mediated through the inhibition of PARP-1.

These experiments were extended to polarized CFBE41o^−^ cells that overexpress ΔF508 CFTR. Figures 2D–G shows short-circuit current responses to forskolin and genistein and sensitivity to the CFTR inhibitor (CFTRinh-172) after monolayers had been incubated with 1 nM ABT-888 for 24 h. ABT-888 increased the short-circuit current response to forskolin and genistein by almost double compared to that of vehicle controls (Figures 2D,E). This was approximately half the rescue elicited by VRT325 (Figure 2F), and much less than that produced by low temperature pretreatment (Figure 2G) as reported previously for other correctors (Robert et al., 2010). The results under each condition are summarized in Figure 2H. Thus, ABT-888 causes partial rescue of the CFTR-dependent short-circuit current response, which is 6.7% of that measured after low temperature incubation and 40% of that induced by the well studied corrector VRT325.

### Decreasing PARP-1 activity *in vivo* restores chloride activity

**Figure 3:**
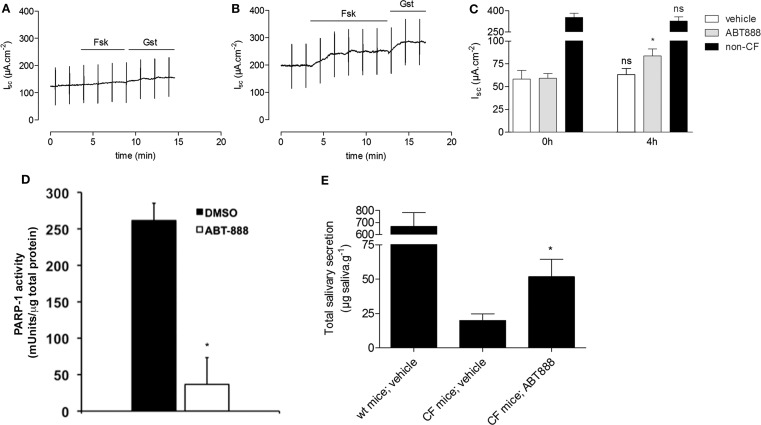
**PARP inhibition partially restores the activity of mouse ΔF508 CFTR**. **(A-B)** Representative short-circuit current (*I*_sc_) traces from mouse ileum from ΔF508-CFTR homozygous mice and non-CF control littermates. 10 μM forskolin (Fsk) and 50 μM genistein (Gsk) were added before [**(A)**, time 0 h] and after [**(B)**, 4 h] incubation with 1 nM ABT-888. **(C)** Bar graph showing the change in short-circuit current (Δ*I*_sc_) after adding Fsk + Gst. *I*_sc_ responses were measured using 2–3 pieces of ileum from each mouse before (0 h) and after (4 h) pre-treatment with vehicle (*N* = 5 mice), or ABT-888 (*N* = 5 mice). Data are presented as the mean ± SEM; relative to their respective controls at time 0 h, calculated using a paired *t*-test; *P < 0.05. Note break in axis. **(D)** PARP-1 activity in vehicle-treated ileum (*N* = 5 mice) and ABT-888 treated ileum (*N* = 5 mice) after 4 h incubation. Data are expressed as mean ± SEM, and significance was calculated by paired *t*-test; *P < 0.05. **(E)** ABT-888 partially restores salivary secretion in mouse salivary glands. Total saliva secreted by homozygous ΔF508 CFTR mice after daily intraperitoneal injection with vehicle (saline; *n* = 5) or ABT-888 (*n* = 5) for 2 days. Means ± SEM; *P < 0.05 by a paired Student’s *t*-test.

To determine if PARP-1 inhibitors are effective in other tissues, mouse ileum was mounted in Ussing chambers and treated *ex vivo* with 1 nM ABT-888 for 4 h. Short-circuit current responses to forskolin and genistein were measured using pieces of ileum dissected from ΔF508 CFTR homozygotes and non-CF littermate controls. These were first taken at time 0 (Figure 3A), and then measured after 4 h incubation with 1 nM ABT-888 (Figure 3B) or saline alone (Figure 3B). Incubation with ABT-888 for 4 h increased the response to forskolin + genistein by ∼30% relative to that measured at time 0 (Figure 3C, *N* = 5, Students’ *t*-test, *P* < 0.05). Incubation in saline alone for 4 h did not alter the *I*_sc_ response to forskolin + genistein (Figure 3C). The increase observed after this relatively brief (i.e., 4 h) exposure to ABT-888 indicates restoration of ∼7% of the wild type current response. PARP-1 activity in mouse ileum was measured by ELISA immediately after experiments with and without ABT-888. Incubation with ABT-888 for 4 h inhibited 85% of the PARP-1 activity measured in parallel experiments in control saline (Figure 3D, *n* = 3, Student’s *t*-test, *P* < 0.05) confirming that it is absorbed rapidly and is effective in native tissue (Muscal et al., 2010).

Studies were also carried out to assess *in vivo* correction in ΔF508 mice (van Doorninck et al., 1995). ABT-888 (5 mg/kg/day) in saline was given daily by intraperitoneal injection; 3 mg/kg/day dose has been shown previously to inhibit PAR activity *in vivo* (Donawho et al., 2007) and results were compared with saline alone. β-adrenergic stimulated salivary secretion was measured by subcutaneous injection of isoprenaline into the cheek after blocking cholinergic stimulation with atropine (Best and Quinton, 2005). ABT-888 injections increased the saliva secretion response to isoprenaline 2.6 times when compared with untreated CF mice (Figure 3E, *P* < 0.05). This corresponds to 7.8% of the response of wild type mice (51.8 ± 2.4 μg g^−1^ vs. 667.5 ± 4.2 μg g^−1^; Figure 3E).

### PARP-1 inhibition promotes ΔF508 CFTR maturation

**Figure 4:**
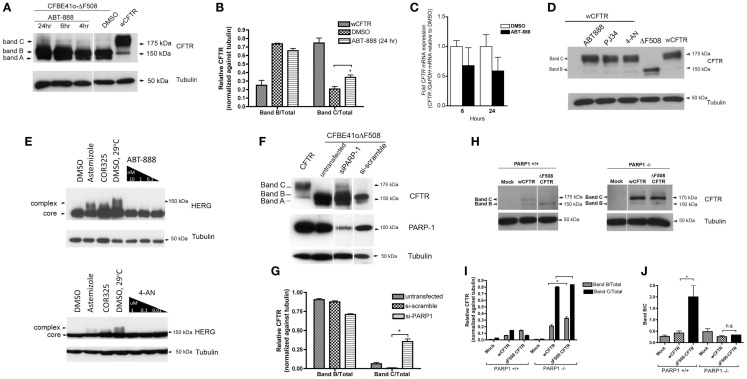
**PARP-1 pharmacological inhibition and genetic deletion promotes the trafficking of ΔF508 CFTR in CFBE41o^−^ cells**. **(A)** CFBE41o^−^ cells expressing ΔF508 CFTR were treated for the indicated times with 1 nM ABT-888 and immunoblotted against CFTR to monitor increases in complex glycosylation (band C). Note the appearance of band C with ABT-888 treatment and an increase in band B. Band A is also detected (ABT-888 and DMSO). **(B)** Mean ± SD ratios of the densitometry signals for band B/total and band C/total, normalized against tubulin. *P < 0.05 (*n* = 3, *t*-test). **(C)** To rule out transcriptional up-regulation by ABT-888, the relative expression of *CFTR* mRNA in CFBE41o^−^ cells (not overexpressing CFTR) was quantified by qRT-PCR. Although there is a trend toward a decrease in *CFTR* mRNA with ABT-888 treatment at the indicated times it does not reach statistical significance. Results are reported as mean ± SD, *P* = *n.s* (*n* = 3, *t*-test). **(D)** PARP-1 inhibitors do not increase the trafficking of wild type CFTR overexpressed in CFBE41o^−^ cells and **(E)** ABT-888 treated HEK 293 cells overexpressing the mutant HERG (G601S) does not increase the trafficking of the ER-retained HERG mutant G601S. We also tested 4-AN at the indicated concentrations in the same system and found no effects suggesting specificity of ABT-888 for the ΔF508-CFTR mutant. **(F)** PARP-1 silencing by siRNA partially restores the trafficking of ΔF508 CFTR in CFBE41o^−^ (overexpressing ΔF508) cells. Immunoblot analysis of CFTR following siRNA-mediated silencing of PARP-1 (48 h). PARP-1 knockdown was monitored by immunoblotting against PARP-1; PARP-1 expression was significantly reduced compared to si-scramble but was not completely abrogated. Note the appearance of band C in siPARP-1 and an increase in band B. **(G)** Mean ± SD ratios of the densitometry signals for band B/total and band C/total, normalized against tubulin. *P < 0.05 (*n* = 3, *t*-test). **(H)** Complete deletion of *PARP-1* restores ΔF508 trafficking. Mouse embryonic fibroblasts lacking PARP-1 (PARP1^−/−^) were transfected with wild type CFTR or ΔF508 CFTR in pCDNA3.1 (and empty plasmid, MOCK). The cells were lysed and analyzed by immunoblotting with anti-CFTR (23C5, 1:20 dilution). Most detectable CFTR migrates at 175 kDa, suggesting the complex glycosylated form of ΔF508 CFTR is predominant in cells lacking PARP-1. When MEF cells containing PARP-1 (PARP1^+/+^) are transfected with same constructs the level of CFTR expression was much lower overall no band C is detected in ΔF508. **(I)** Mean ± SD ratios of the densitometry signals for band B/total and band C/total, normalized against tubulin. *P < 0.05 (*n* = 3, *t*-test) **(J)** band B/C ratios indicate more band B, as expected in the PARP-1^+/+^ cells for ΔF508 CFTR, while in the PARP-1^−/−^ cells there is no difference between the wCFTR and ΔF508 CFTR transfections, suggesting that most of the CFTR is in the mature band C form. *P < 0.05 (*n* = 3, *t*-test).

Since we observed an improvement in chloride channel activity levels with PARP-1 inhibition, we assume that ΔF508 had matured beyond the ER. To examine this further we monitored the maturation of ΔF508 CFTR by immunoblotting CFBE41o^−^ cells (overexpressing ΔF508 CFTR) that had been treated with 1 nM ABT-888. After 6 h we observed a band migrating at 175 kDa that may be complex glycosylated ΔF508 CFTR (band C; see Figure 4A). We also observed an increase in the core-glycosylated (band B) form of ΔF508 CFTR (Figure 4A). We quantified the relative amounts of each band by densitometry and found a significant increase in the fraction of mature CFTR (band C/total) treated with ABT-888 (0.34 ± 0.04) when compared with DMSO (0.20 ± 0.05), Figure 4B (*P* = 0.02). We ruled out transcriptional effects of ABT-888 by monitoring the relative abundance of *CFTR* mRNA at 6 and 24 h treatment with ABT-888. When CFBE41o^−^ cells overexpressing ΔF508 CFTR were treated with ABT-888 (1 nM), real-time PCR revealed no change in CFTR mRNA levels after both 6 and 24 h exposure (Figure 4C, *P* = n.s). To test for specificity of ABT-888, we monitored the maturation of wild type CFTR in CFBE41o^−^ cells (exogenously expressing wt-CFTR) treated with ABT-888 and two other PARP-1 inhibitors, PJ34 and 4-AN by immunoblotting (Figure 4D). However we found no effects on the steady-state levels of the immature or mature glycoforms of wild type CFTR, suggesting that PARP-1 inhibition targets the ΔF508 CFTR mutant specifically. The specificity of PARP-1 inhibition was assessed by comparing the ability of ABT-888 and 4-AN to correct the mutant form of the human ether-à-go-go-related K^+^ channel (hERG: hERG G601S) expressed in HEK 293 cells, which is also retained in the ER (Figure 4E). PARP-1 inhibition did not improve the processing of the mutant form of hERG (Figure 4E) whereas VRT-325 increased the amount of processed hERG G601S (Figure 4E). Artemizole and low temperature incubation (29°C) are known to correct G601S and are shown as positive controls (Figure 4E). The different selectivities of VRT-325 and the PARP-1 inhibitors also suggest they act through distinct pathways.

To determine if the trafficking correction observed is indeed PARP-1 dependent we examined the effect of silencing PARP-1 in CFBE41o^−^ cells that overexpress ΔF508 CFTR. siRNA-mediated silencing of PARP-1 (which accounts for 85–90% of the pADPr protein in mammalian cell; Pacher and Szabo, 2008) reduced PARP-1 protein expression as expected (Figure 4F), and this was accompanied by the appearance of some complex glycosylated (band C) ΔF508 CFTR, indicating escape from the ER (Figure 4F). We quantified the relative amounts of band C and band B by densitometry and found a significant increase in the mature form of CFTR upon silencing of PARP-1 by siRNA (0.35 ± 0.04) relative to si-scramble and/or untransfected (Figure 4G) when normalized to tubulin. To extend this to a cell system that is devoid of PARP-1, we carried out experiments with PARP-1^−/−^ mouse embryonic fibroblasts (MEFs) that had been transfected with triple-HA tagged Δ508 CFTR (Figure 4H). ΔF508CFTR-3HA was found almost exclusively in the complex glycosylated CFTR (band C) form (Figure 4H) confirming that the effect of PARP-1 inhibitors on the trafficking of ΔF508 is mediated by PARP-1 and is not an off-target drug effect. We quantified the relative amounts of immature and mature forms of CFTR normalized against tubulin in PARP-1^−/−^ MEF cells (Figure 4I) and found there was no significant difference in the amount of mature CFTR in ΔF508 transfected PARP-1^−/−^ MEFs vs. wCFTR transfected cells (Figure 4I, *P* = n.s.). There was more immature CFTR (band B) in the ΔF508 CFTR transfected cells even when corrected for loading with tubulin (Figure 4I, *P < 0.05).

In MEF cells expressing PARP-1 (PARP-1^+/+^) the complex glycosylated (band C) form of CFTR was only detected when wild type CFTR was transfected, whereas ΔF508CFTR was found predominantly in the core-glycosylated (band B) form (Figures 4H,I) as expected. Particularly striking is the increase in the expression of CFTR in the absence of PARP-1^−/−^ (Figure 4I, *P* < 0.05). We calculated the ratio of band B/C (Figure 4J), which we expect to be higher in the ΔF508 CFTR transfected PARP-1^+/+^ cells. While in the PARP-1^−/−^ transfected cells there is no difference between the band B/C ratio in wCFTR and ΔF508 transfected cells (Figure 4J, *P* = n.s.), in the PARP-1^+/+^ cells the band B/C ratio for ΔF508 transfections is five times higher (2.0 ± 0.7 for ΔF508 CFTR vs. 0.41 ± 0.14 for wCFTR, Figure 4J, *P* < 0.05).

### Increasing PARP-1 activation with peroxynitrite inhibits the trafficking of wild type CFTR

**Figure 5:**
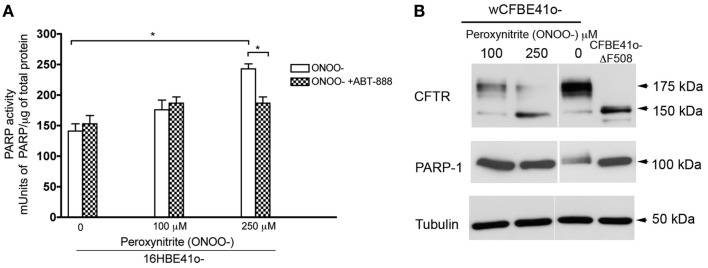
**Increasing PARP-1 activity decreases trafficking of wild type CFTR**. **(A)** 16HBE14o^−^ (non CF) were treated with the indicated doses of peroxynitrite (ONOO^−^), a potent PARP-1 activator. PARP-1 activity was monitored by ELISA. PARP-1 significantly increased at 250 μM ONOO^−^ (3 h treatment). Blocking PARP-1 with ABT-888 abolished the effects of peroxynitrite on PARP-1 activation suggesting that the effects we see are PARP-1 mediated. Bar graph represents the mean ± SEM (*n* = 3, with three replicates each). Statistical significance was determined using a paired *t*-test; *P < 0.05. **(B)** To monitor trafficking *decreases*, CFBE41o^−^ cells stably overexpressing wild type (wt) treated with the indicated concentrations of ONOO^−^ for 3 h. Lysates from CFBE41o^−^ cells overexpressing ΔF508 CFTR are also shown. A decrease in trafficking promoted by ONOO^−^ is observed at 250 μM ONOO^−^ with a decrease in complex glycosylated CFTR. No cleavage of PARP-1 was observed.

Since reducing PARP-1 activity restores mutant CFTR trafficking and function, we hypothesized that increasing PARP-1 activation should reduce trafficking. To test this hypothesis and monitor trafficking decreases, CFBE41o^−^ cells expressing heterologous wild type CFTR was exposed to the physiologically relevant PARP-1 activator peroxynitrite (Szabo et al., 2007). PARP-1 activity was increased by acute peroxynitrite treatment (3 h) with 100 and 250 μM, reaching a maximal two-fold increase at 250 μM (Figure 5A). This inhibition was partially blocked by ABT-888, confirming that the stimulation by peroxynitrite occurs through the activation of PARP-1 (Figure 5A).

Treating CFBE41o^−^ cells overexpressing wild type CFTR with 250 μM peroxynitrite caused a marked reduction in complex glycosylated CFTR (Figure 5B), Increasing PARP-1 activity also increased the retention of core-glycosylated CFTR in the ER (Figure 5B). There was also some reduction in total CFTR protein expression at 100 and 250 μM. This reduction was apparently not due to apoptosis since cleavage of PARP-1 into 85 and 100 kDa fragments, a hallmark of apoptosis, was not observed (Figure 5B). We also observed increased PARP-1 expression in cells treated with 100–250 μM peroxynitrite (compared to untreated wild type cells), which reached levels that were comparable to those in CFBE41o^−^ cells overexpressing ΔF508 CFTR (Figure 5B).

Overall, our data suggests interdependence between the levels of oxidative stress, PARP activation and CFTR biogenesis. Taken together, we have shown in several CF models, including human primary bronchial epithelial cells (Figure 1B) and in CF cells (that do not overexpress ΔF508, Figure 1C), that PARP-1 activity is elevated when compared to matched non-CF cells. This suggests oxidative stress caused by a misfolded protein, leading to increased PARP-1 activity.

Decreasing the levels of PARP-1 activity by treatment with PARP-1 inhibitors or the absence of PARP-1, results in a partial restoration of ΔF508 CFTR trafficking and its function.

## Discussion

High PARP-1 activation in response to oxidant-mediated DNA damage is an important pathway during tissue injury (Pacher and Szabo, 2008). In this study we considered the modulation of PARP-1 activity by pharmacological inhibition and genetic silencing or deletion and how this affects CFTR function and expression. Physiologically relevant levels of DNA damage and PARP-1 activation have been demonstrated in pulmonary diseases, such as asthma, acute lung injury, and COPD (42–45), however these have not been investigated in CF. In view of the central role it plays in cellular stress responses (Luo and Kraus, 2005) and reports of exaggerated inflammation (Galli et al., 2012), elevated oxidative stress (Galli et al., 2012), and metabolic dysregulation in CF patients (Wetmore et al., 2010), we hypothesized that PARP-1 might influence CFTR biology.

We observed higher PARP-1 activity in HBEs derived from patients homozygous for ΔF508 CFTR than in HBEs from non-CF donors, and similarly higher activity in the CFBE41o^−^ cell line compared to the non-CF line 16HBE14o^−^. To our knowledge this is the first evidence that PARP-1 is upregulated in CF. Although there may be many differences between the CFBE41o^−^ and 16HBE14o^−^ cell lines, e.g., in the functional expression of drug-transporter P-gp assayed by Rhodamine123 (Ehrhardt et al., 2006), a major distinguishing feature is the presence of a misfolded and dysfunctional CFTR channel in CFBE41o^−^. Further evidence that the difference in PARP-1 activity is due to the presence of ΔF508 CFTR comes from the results with primary cells from patients. Despite the intrinsic variability between different patients, the same pattern was observed, i.e., PARP-1 was higher in cells from both CF patients compared to non-CF subjects. Nevertheless, the relationship between CFTR and PARP-1 activity will need to be extended to a larger cohort in the future.

Since PARP-1 is a DNA damage sensor, one might expect its activity in CF cells to reflect increased DNA damage. Indeed, DNA fragmentation has been reported in intestinal cells from CF patients (Maiuri et al., 1997), and elevated levels of oxidative stress markers and DNA damage have also been reported in CF (Brown et al., 1995, 1996). CF patients present with several abnormalities in oxidative stress, including elevated ROS generation, a constitutive defect in glutathione metabolism, and reduced intake of fat-soluble antioxidant vitamins (Galli et al., 2012), some of which are endogenous or natural PARP-1 inhibitors (Banasik et al., 1992). Aside from nicotinamide, natural occurring inhibitors of PARP-1 include tryptophan-related compounds, purines, unsaturated fatty-acids (including linoleic acid and arachidonic acid), and carotenoids (Banasik et al., 1992) the levels of which are reportedly low in CF patients (Wetmore et al., 2010, Galli et al., 2012).

We observed a correlation between PARP-1 activity and CFTR, consistent with reports that (1) CFTR dysfunction itself can lead to oxidative stress (Bartoszewski et al., 2008; Chen et al., 2008), (2) ROS reduce wild type CFTR protein expression and cAMP-mediated Cl^−^ secretion in airway epithelia (Bebok et al., 2002), and (3) ER retention of CFTR may contribute to inflammation (Rottner et al., 2009).

PARP-1 activity was modulated by treating CF epithelial cells with the potent PARP-1 inhibitor ABT-888 (Veliparib) at low concentrations, lower than needed to observe maximal inhibition of PARP-1 (maximum inhibition at 1 nM vs. IC_50_ = 5 nM). The reason for this extraordinary potency remains unknown, however it was observed in several cell types (Figures 1–3). ABT-888 inhibition of PARP-1 activity was variable among different cell types, consistent with previous reports (Virag, 2005). For example, inhibition was stronger in mouse ileum (Figure 3D). Although ABT-888’s potency as a ΔF508 corrector has been evaluated in recombinant cell lines and model systems it will be important to investigate its action further in primary cells. Based on the previous reports of correctors that work modestly *in vitro* (Pedemonte et al., 2010) not advancing further into pre-clinical or human CF trials for lack of specificity, off-target effects and/or insufficient levels of restoration of trafficking (Pedemonte et al., 2010) highlights the importance of assessing correctability in primary cells. We measured the effects of ABT-888 treatment on ΔF508 CFTR function in native HBEs derived from a single patient by Ussing chamber measurements of chloride activity and while we found a modest increase in activity, this was not statistically significant (data not shown). However, there is a reported large variation in the “correctability” by a single compound in different patients (the short current response of CF primary lung bronchial epithelial cells to VX-809 varied between 3.4–14.9% of non-CF donor lungs; Van Goor et al., 2011). More patients will need to be tested. Additionally, we also predict that there will be variability in the levels of oxidative burden and consequently PARP-1 activation between patients. A larger cohort will have to be tested to address this.

Finally, peroxynitrite activated PARP-1 and reduced the maturation of wild type CFTR (Figures 5A,B), and this effect was blocked by ABT-888, strongly suggesting that peroxynitrite was acting through PARP-1. Although the activation of PARP-1 by peroxynitrite and its involvement in disease is well documented (Pacher and Szabo, 2008), peroxynitrite is a potent oxidant and we cannot exclude other potential mechanisms of action (Figure 5A). Previous studies have demonstrated that (1) oxidants affect CFTR function (Rottner et al., 2009), (2) CFTR dysfunction itself may lead to oxidative stress (Chen et al., 2008), (3) oxidative stress suppresses CFTR expression (Cantin et al., 2006; Bartoszewski et al., 2011), and (4) increases in reactive oxygen nitrogen species may decrease wild type CFTR protein expression and cAMP-mediated Cl^−^ secretion by airway epithelia (Bebok et al., 2002).

ΔF508 CFTR maturation was dramatically altered in PARP-1 knockout cells. Only the mature glycoform was detected in PARP-1 null MEFs (Figure 4H). This is consistent with the partial restoration of ΔF508 trafficking in CFBE41o^−^ cells (Figures 4A,B) when PARP-1 activity was inhibited pharmacologically or silenced by RNA interference (Figures 4F,G). It has been shown that PARP-1 knockout mice display altered expression of redox-sensitive, AP-1-dependent genes, proinflammatory mediators, and heat shock proteins (including HSP70; Andreone et al., 2003) known to regulate CFTR gene expression, folding or function (McCarthy and Harris, 2005). Moreover, the PARP-1 knockout mouse is also resistant to various models of inflammation, the mechanism of which occurs via deficient NF-κB activation (Schreiber et al., 2006), which requires PARP-1 as a co-activator.

The effects of PARP-1 inhibition seem to be specific for the mutant CFTR form, as we do not observe any improvements in the processing of the wild type CFTR (Figure 4D) nor for the mutant form of the human ether-à-go-go-related K^+^ channel (hERG: hERG G601S) expressed in HEK 293 cells, which is also retained in the ER (Figure 4E).

These results suggest that the effects we observe are ΔF508 CFTR specific and are linked to elevated oxidative stress, which is not elevated in the wild type CFTR cells. We propose that restoring homeostasis through the attenuation of PARP-1 activity increases translation and proteostasis at least partly because oxidative stress is lowered. Although the mechanism of action is yet undetermined, we hypothesize that PARy(lation) of key members of the CFTR folding interactome such as the HSP90 co-chaperone Hop, among others (Gagne et al., 2008), may alter their expression and function promoting folding, and altering interactions with partners, consistent with a rapid response (Figure 4A). PARP-1’s role in intracellular trafficking is expanding as recently reviewed (Abd Elmageed et al., 2011) highlighting the multiple roles and pleiotropic effects of PARP related pathways.

Achieving therapeutically-relevant trafficking of ΔF508 CFTR *in vivo* may require a combination of two or more corrector drugs, and may also require antioxidant therapies due to underlying defects in the regulation of oxidative stress and inflammation. The present results suggest that addressing the trafficking and oxidative stress problem through manipulation of PARP-1 and related pathways may be a useful approach for restoring homeostasis and should be investigated further in the context of CF therapeutics.

## Conflict of Interest Statement

The authors declare that the research was conducted in the absence of any commercial or financial relationships that could be construed as a potential conflict of interest.
